# Perceived commuting stress, residential nighttime light, and mental health: an environmental stress perspective

**DOI:** 10.3389/fpsyg.2026.1817776

**Published:** 2026-08-17

**Authors:** Haoxian Zhang, Yifei Meng, Hongyu Du, Xiaobo Feng, Yanqing Xu

**Affiliations:** 1 College for Elite Engineers, China University of Geosciences, Wuhan, Hubei, China; 2 Gansu Federation of Trade Unions, Lanzhou, Gansu, China; 3 School of Remote Sensing and Information Engineering, Wuhan University, Wuhan, Hubei, China; 4 Institute of Ecology and Sustainable Development, Shanghai Academy of Social Sciences, Shanghai, China; 5 Department of Pain Medicine, Zhongnan Hospital of Wuhan University, Wuhan, Hubei, China

**Keywords:** commuting stress, environmental stressors, mental health, nighttime light, perceived stress, stress appraisal

## Abstract

Daily environmental stressors embedded in urban life may influence mental health not only through direct exposure but also through the ways individuals perceive and respond to these demands. Among these stressors, commuting represents a recurrent daily burden whose psychological impacts may vary according to the surrounding residential environment. This study integrates satellite-derived nighttime light (NTL) exposure with individual-level survey data to examine the relationships among objective commuting conditions, perceived commuting stress, and mental health outcomes, as well as the moderating role of residential nighttime environments. Using data from 7,093 working adults in Jiayuguan City, China, we conceptualize commuting stress within a stress-process framework that distinguishes objective commuting conditions from subjective stress appraisal. A Perceived Commuting Stress Index (PCSI) was developed to capture integrated psychological responses to commuting experiences. Multivariable regression and structural equation modeling showed that objective commuting characteristics were associated with perceived stress, depressive symptoms, and anxiety primarily through perceived commuting stress, highlighting the central mediating role of subjective appraisal. Residential NTL exposure showed limited direct associations with mental health outcomes but strengthened the association between commuting stress and both perceived stress and depressive symptoms. These patterns were consistent with the possibility that nighttime urban environments may interfere with recovery-related processes under elevated commuting burden. The findings illustrate how satellite-derived NTL exposure can contribute to mechanism-oriented urban health research by capturing environmental contexts relevant to stress and mental health.

## 1 Introduction

Environmental stressors embedded in urban daily life have increasingly been recognized as important determinants of mental health. Psychological stress is often not solely triggered by major life events but rather emerges through prolonged and repeated exposure to everyday environmental stressors that accumulate gradually over time. Within contemporary urban settings, commuting represents one of the most persistent and unavoidable forms of such daily environmental stress. Characterized by repetitiveness, long-term continuity, and limited individual control, commuting extends beyond a mere travel behavior and may function as a chronic environmental stressor in everyday urban life (Gottholmseder et al., 2009). A growing body of evidence suggests that adverse commuting conditions are associated with elevated perceived stress, increased depressive symptoms, and poorer mental health outcomes, particularly among the urban working population (Qiu et al., 2024; Zhang and Ma, 2024).

Existing commuting studies have primarily conceptualized commuting stress through objective indicators such as commuting time, travel distance, or travel mode. Although these measures capture certain physical characteristics of commuting demands, they provide limited insight into how commuters psychologically experience and interpret these demands within complex urban environments. Empirical findings based solely on objective indicators have remained inconsistent. Some studies have reported that longer commuting duration significantly reduces subjective well-being (Stutzer and Frey, 2008), whereas others have found that such associations weaken or even disappear after controlling for socioeconomic characteristics (Chatterjee et al., 2020). These inconsistencies suggest that the psychological consequences of commuting may depend not only on objective exposure itself, but also on how individuals cognitively appraise and emotionally respond to commuting-related environmental demands.

This perspective aligns with the environmental stress appraisal framework, which posits that stress responses arise from individuals’ cognitive evaluation of environmental demands rather than from environmental stimuli alone. The effects of environmental stressors are mediated through subjective perception, emotional regulation, and coping processes (Lazarus and Folkman, 1984). Even under similar commuting conditions, the degree of psychological burden experienced by individuals may vary substantially depending on their perceptions of crowding, uncertainty, physical discomfort, or loss of control during travel. High-density commuting environments may intensify psychological tension, while uncertainty associated with traffic congestion and travel delays may further amplify anxiety responses (Conceiçao et al., 2023; Evans and Wener, 2007). These subjective experiences constitute a critical psychological pathway linking objective commuting conditions to mental health outcomes.

Importantly, the psychological resources depleted during daily commuting and work-related activities require restoration through opportunities provided by the residential environment after these activities conclude (Chatterjee et al., 2020; Hartig et al., 1991). In environmental psychology, recovery from everyday environmental demands is understood not merely as the absence of stressors, but as a process through which individuals regain depleted emotional, cognitive, and physiological capacities (Hartig et al., 1991; Kaplan, 1995; Ulrich et al., 1991). As a key restorative setting, the residential environment not only provides physical shelter but also facilitates emotional relief, psychological detachment, and physiological recovery following stressful daily activities (Van den Berg et al., 2010; White et al., 2019).

However, within the contemporary urban nighttime environment, such restorative opportunities may be constrained by persistent socioeconomic activities, continuous artificial illumination, and prolonged environmental stimulation. These environmental characteristics maintain elevated levels of sensory arousal during periods typically associated with rest and psychological recovery, thereby undermining individuals’ ability to mentally detach from daytime demands (Evans and McCoy, 1998; Sonnentag and Fritz, 2007). Among these factors, excessive artificial light exposure at night, particularly blue light, has been shown to disrupt circadian rhythms and suppress melatonin secretion, consequently impairing sleep quality and increasing the risk of anxiety (Chepesiuk, 2009; Falchi et al., 2011; Shao et al., 2025). Sleep disruption, in turn, directly diminishes individuals’ capacity to cope with subsequent stressors (Palmer et al., 2024). Therefore, the urban nighttime environment should not be regarded merely as a passive residential setting; rather, the restorative conditions it provides may be related to how commuting-related stress is experienced and perceived across daily life.

To objectively characterize this stress context, satellite-derived NTL data have emerged as an important approach for representing urban nighttime environments. Beyond capturing physical illumination intensity, NTL data also reflect objective characteristics of urbanization level, socioeconomic activity intensity, and the continuity of human activities, thereby capturing the temporal extension of the modern “24-h society” (Henderson et al., 2012; Levin and Duke, 2012; Levin et al., 2020). Compared with traditional questionnaire-based measures that rely on respondents’ retrospective recall, NTL data provide continuous, large-scale, and spatially explicit representations of environmental exposure, effectively reducing subjective recall bias. The increasing availability of high-resolution NTL imagery further enables more refined assessments of residential nighttime environmental conditions at the individual level (Zheng et al., 2023). Although previous studies have linked NTL exposure to a range of adverse mental health outcomes, findings regarding its direct association with mental health remain inconsistent after controlling for confounding environmental factors such as air pollution and urban density (Helbich et al., 2024; Paksarian et al., 2020). These inconsistent findings suggest that the influence of NTL on mental health may not operate primarily as a stable direct effect, but rather may depend on individuals’ ongoing processes of daily stress exposure and recovery.

Despite growing attention to commuting stress and urban nighttime environments, three major gaps remain in the existing literature. First, studies on commuting have relied excessively on objective indicators such as commuting duration and travel distance, while paying insufficient attention to processes of subjective stress perception. As a result, they provide limited insight into how objective exposures are associated with health outcomes through processes of subjective appraisal. Second, research examining commuting stress has rarely incorporated the restorative or stress-amplifying characteristics of residential environments into its analytical framework, thereby overlooking the critical role of post-commuting recovery conditions in either the accumulation or alleviation of stress. Third, nighttime environmental exposure has predominantly been investigated as a direct risk factor for mental health, whereas its role as a contextual moderator within everyday stress processes remains poorly understood. In particular, it remains unclear whether intense nighttime urban activity may amplify the adverse effects of commuting stress on mental health by constraining psychological recovery after commuting.

To address these gaps, this study integrates satellite-derived NTL data with individual psychological survey data within the environmental stress appraisal framework and contributes in three key respects. First, a multidimensional commuting stress framework is developed to distinguish objective commuting conditions from subjective stress perception. Based on this framework, a Perceived Commuting Stress Index (PCSI) is constructed to capture individuals’ comprehensive subjective appraisal of commuting-related stress. Second, perceived commuting stress is conceptualized as the proximal mechanism linking objective commuting conditions to mental health outcomes, thereby empirically examining the pathway from “objective exposure–subjective stress–mental health” and advancing the literature from an exposure-oriented perspective toward a mechanism-oriented perspective. Third, high-resolution NTL data are employed to characterize urban nighttime stress contexts at scale and introduced into commuting stress analyses as a contextual moderating factor. This approach not only assesses the direct effects of nighttime environments on mental health but also reveals their role as contextual amplifiers that modify the relationship between commuting stress and mental health. By integrating subjective appraisals of commuting stress with spatially measured characteristics of nighttime residential environments, this study provides an integrated analytical framework for understanding how objective environmental exposures, subjective stress appraisal, and contextual recovery conditions jointly shape mental health outcomes.

Based on the theoretical framework and prior evidence, we propose the following hypotheses:

*H1*: Objective commuting demands are positively associated with perceived commuting stress.

*H2*: Perceived commuting stress is positively associated with perceived stress, depressive symptoms, and anxiety symptoms.

*H3*: Perceived commuting stress mediates the association between objective commuting demands and mental health outcomes.

*H4*: NTL levels in residential areas moderate the association between perceived commuting stress and mental health, such that this association is stronger under higher NTL exposure.

## 2 Materials and methods

### 2.1 Study area and data sources

The data used in this study were derived from a mental health and work environment survey of trade union members conducted in Gansu Province, China. The survey was designed and implemented by the research team, collecting individual-level information on sociodemographic characteristics, commuting behaviors, occupational conditions, lifestyle factors, and mental health status. Eligible participants included legally employed workers from a wide range of occupational sectors, including manufacturing, transportation, education, healthcare, public services, and commerce. All participants were informed of the study objectives and data usage prior to participation, and all questionnaires were completed voluntarily and anonymously. The study adhered to relevant ethical standards, and all collected data were used exclusively for scientific research purposes. The survey was conducted between October and December 2025. During data cleaning, strict quality control procedures were implemented, including the exclusion of questionnaires with unusually short completion times, substantial missing information, or evident response inconsistencies. The final dataset comprised 26,064 valid responses.

This study focuses on a subsample of employed adults in Jiayuguan City (*n* = 7,093). Jiayuguan is a representative industrial city in northwestern China, characterized by a relatively stable labor force, a high proportion of industrial and shift-based employment, and generally regular commuting patterns. In addition, the city exhibits a compact urban form with clearly defined built-up boundaries, resulting in a relatively concentrated spatial distribution of residential and employment areas. NTL exposure in Jiayuguan also shows pronounced spatial heterogeneity across industrial, commercial, and residential zones. Taken together, these characteristics provide a valuable empirical context for examining the relationships among commuting stress, nighttime environmental exposure, and mental health among employed adults.

### 2.2 Measurement of mental health

Depressive symptoms and their severity were assessed using the Patient Health Questionnaire-9 (PHQ-9), which is widely applied in clinical screening, mental health research, and evaluation of treatment outcomes. The PHQ-9 consists of nine items, each rated according to symptom frequency, yielding a total score ranging from 0 to 27, with higher scores indicating more severe depressive symptoms. In the present study, the PHQ-9 demonstrated high reliability (Cronbach’s *α* = 0.928). Anxiety symptoms were measured using the Generalized Anxiety Disorder Scale-7 (GAD-7), a seven-item instrument assessing experiences over the past 2 weeks, with higher scores reflecting greater anxiety. The GAD-7 showed excellent internal consistency in this sample (Cronbach’s *α* = 0.955). Perceived stress was evaluated using the 10-item Perceived Stress Scale (PSS-10), which assesses individuals’ subjective perceptions of uncontrollability, unpredictability, and stress intensity in daily life over the past month, and is a widely recognized tool for measuring everyday stress experiences. The PSS-10 exhibited high reliability in the current sample (Cronbach’s *α* = 0.909).

### 2.3 Construction of commuting stress variables

#### 2.3.1 Objective commuting stress indicators

Objective commuting stress refers to quantifiable and structural commuting constraints encountered by individuals in their daily commuting processes. It primarily captures relatively independent characteristics of commuting that are less influenced by subjective evaluation, including time investment, economic costs, process complexity, and institutional flexibility. Based on the availability of survey data and theoretical relevance, this study constructs an index system of objective commuting stress across four dimensions: time, process, economic, and institutional. Specifically, commuting duration is used to measure the time cost of commuting; the number of transfers reflects the complexity and uncertainty of the commuting process; commuting cost captures the direct financial expenditure associated with commuting; and commuting flexibility describes whether individuals have access to staggered or flexible working hours, with higher values indicate lower commuting flexibility and stronger institutional constraints.

#### 2.3.2 Perceived commuting stress index (PCSI)

Perceived commuting stress reflects individuals’ subjective psychological evaluation of their daily commuting experiences. Drawing on prior research on environmental stress appraisal and commuting-related stress perception, this study captures key psychological dimensions of commuting stress using four indicators: (1) perceived crowding during commuting, representing excessive environmental density and social load; (2) physical discomfort, such as noise or pushing, reflecting sensory and bodily strain; (3) anxiety or irritability induced by commuting, representing emotional stress responses; and (4) perceived uncertainty in commuting time (e.g., traffic congestion), reflecting perceived lack of control over the commuting process. All items were measured using a 5-point Likert scale ranging from 1 (lowest perceived stress) to 5 (highest perceived stress), with higher scores indicating greater subjective stress experienced during commuting.

To assess the construct validity of the Perceived Commuting Stress Index (PCSI), an exploratory factor analysis (EFA) was conducted. The Kaiser–Meyer–Olkin (KMO) statistic indicated good sampling adequacy (KMO = 0.77), and Bartlett’s test of sphericity was significant [*χ*^2^(6) = 8517.582, *p* < 0.001], supporting the suitability of the data for factor analysis. The EFA revealed a unidimensional structure, with all four items exhibiting strong factor loadings (range: 0.689–0.818). The extracted factor explained 64.3% of the total variance, indicating that these items collectively capture a unified construct of perceived commuting stress. Reliability analysis further demonstrated good internal consistency (Cronbach’s *α* = 0.792).

To reduce the influence of differing item scales and distributions, all four items were first standardized using *z*-scores prior to index construction. The standardized scores were then averaged to generate the individual-level commuting stress index (PCSI), with higher scores indicating greater perceived commuting stress. Compared with single-item subjective measures, the PCSI provides a more comprehensive representation of the multidimensional psychological evaluation of commuting-related stress. In subsequent analyses, the PCSI is used both as a key explanatory variable and as a mediating variable linking objective commuting demands to mental health outcomes within a structural equation modeling framework.

### 2.4 Satellite-derived NTL exposure and spatial linkage

The NTL exposure in residential areas was estimated using NTL imagery acquired in October 2025 by the Glimmer (GLI) sensor onboard the Sustainable Development Goals Satellite 1 (SDGSAT-1). The temporal coverage of the imagery closely aligns with the survey period, thereby improving temporal consistency between environmental exposure assessment and individual-level survey data. In this study, the panchromatic high-gain band (444–910 nm) with a spatial resolution of 10 m was used to quantify NTL intensity surrounding participants’ residential locations. Residential NTL exposure was conceptualized as a spatial proxy for the intensity of nighttime environmental stimulation and urban activity in the surrounding residential context. This study utilized the SDGSAT-1 GLI Level-4A product, which provides geometrically corrected NTL imagery. Radiometric calibration was conducted as shown in Equation 1:

L=DN×Gain+Bias (1)

In the formula, *L* represents the radiance (W/m^2^/sr/μm), *DN* denotes the band value after relative radiometric calibration, and Gain and Bias are calibration coefficients for gain and offset, respectively. The bias term refers to the average dark current under new moon conditions over ocean scenes. The calibration coefficients are provided by SDG-SAT1; for the high-gain band, the Gain is 0.0000643698, and the Bias is 0.0000183897.

After radiometric calibration, residential addresses of participants were geocoded into geographic coordinates using the Baidu Geocoding API service. All geocoded locations were subsequently subjected to manual map inspection and visual verification to ensure positional accuracy. Residential records that could not be reliably geocoded or spatially validated were excluded from the spatial analysis. In addition, prior to exposure extraction, NTL imagery was visually inspected to remove scenes affected by obvious cloud contamination or imaging artifacts.

Based on previous literature, residential NTL exposure was calculated across multiple spatial scales, including the residential pixel (0 m) and buffers of 50 m, 100 m, 200 m, and 500 m surrounding each residence to assess the robustness of associations across different neighborhood contexts. Smaller buffers better approximate direct individual exposure, whereas larger buffers capture broader neighborhood environmental context. Accordingly, the 500 m buffer was used as the primary analytical scale, while smaller buffers were employed for sensitivity analyses (Browning and Lee, 2017).

### 2.5 Covariates

To reduce potential confounding effects, a range of demographic and socioeconomic variables were included as covariates in the regression models. These variables included gender (male/female); age (<25, 25–34, 35–44, 45–54, and ≥55 years), education level (below high school, college, bachelor’s degree or above), marital status (married, single/divorced/widowed), and number of children were included as categorical variables.

For socioeconomic characteristics, monthly income served as a proxy for income level and was included as a grouped variable (<¥5,000, ¥5,000–8,000, and >¥8,000). Household registration type (local vs. non-local) was also included to control for potential institutional and socioeconomic disparities. Regarding occupational characteristics, years of work experience (continuous variable, in years) reflected differences in career stage; job type (e.g., managerial, technical) was included to account for variations in job responsibilities and work-related stress; and employment type (formal vs. informal) captured differences in employment stability. All covariates were consistently controlled for in the main effect models, mediation analyses, and interaction effect analyses to enhance the robustness of the findings and reduce potential confounding in estimating the commuting stress–mental health relationship.

### 2.6 Statistical analysis

The study employed a multi-step analytical framework to examine the associations among commuting stress, residential NTL exposure, and mental health outcomes, as well as their underlying psychological mechanisms. The primary analyses used the 500 m buffer as the core spatial scale for residential NTL exposure, while additional buffers were included in sensitivity analyses to assess the robustness of the associations across different spatial contexts. Given that some variables deviated from normal distributions, Spearman rank correlation analysis was conducted to assess the bivariate associations between commuting stress indicators and mental health outcomes. The results were visualized using correlation matrices and heatmaps. In addition, Mantel tests were applied to evaluate the overall association structure between the set of commuting stress indicators and the set of mental health variables. Euclidean distance matrices were computed for each variable set, and Mantel’s r statistic was estimated using permutation-based significance testing (Mantel, 1967).

The main effects of commuting stress on mental health were assessed using regression analyses. Regression coefficients and corresponding 95% confidence intervals were reported after sequential adjustment for sociodemographic, socioeconomic, and occupational covariates. Structural equation modeling (SEM) was employed to examine the mediating role of the PCSI in the association between objective commuting demands and mental health outcomes. All SEM results reported standardized path coefficients. Indirect effects were evaluated using a bootstrap approach with 5,000 resamples, and bias-corrected 95% confidence intervals were provided. The moderating role of NTL exposure was tested using interaction models. In these models, an interaction term between the commuting stress index and NTL exposure was included, and the analysis assessed whether NTL exposure altered the strength of the association between commuting stress and mental health after controlling for main effects and covariates. All statistical analyses were performed in R (version 4.3.2), with a statistical significance threshold set at *p* < 0.05.

## 3 Results

### 3.1 Descriptive statistics

The final analytical sample consisted of 7,093 employed adults. Tables 1, 2 present descriptive statistics for sociodemographic characteristics, commuting conditions, mental health outcomes, and residential NTL exposure. The sample was predominantly composed of middle-aged male workers, with most participants being married (81.26%) and holding local urban Hukou (76.09%). Mean scores for depressive symptoms (PHQ-9), anxiety symptoms (GAD-7), and perceived stress (PSS-10) indicate moderate variation in mental health status among participants.

**Table 1:** Sample characteristics and mental health outcomes (*N* = 7,093).

Variables	Frequency	Mean (SD)/proportion
PHQ-9 score	7,093	8.43 (5.73)
GAD-7 score	7,093	6.24 (5.01)
PSS-10 score	7,093	15.56 (7.49)
Age (%)		
<25	209	2.95
25–34	1713	24.15
35–44	2,898	40.86
45–54	1794	25.29
≥ 55	479	6.75
Gender (%)		
Female	1,291	18.20
Male	5,802	81.80
Marital status (%)		
Married	5,764	81.26
Single, divorced or widowed	1,329	18.74
Hukou-based residential status (%)		
Local urban	5,397	76.09
Migrant urban	1,696	23.91
Education (%)		
Below high school	1,699	23.95
Junior college	3,031	42.73
Bachelor’s degree or above	2,363	33.31
Monthly income (%)		
<¥5,000	2,383	33.60
¥5,000–¥8,000	4,101	57.82
>¥8,000	609	8.58
Number of children (%)		
0	1,434	20.22
1	3,965	55.90
≥2	1,694	23.88
Employment type (%)		
Permanent employee	6,586	92.85
Non-permanent employee	507	7.15
Job position type (%)		
Managerial	583	8.22
Technical	928	13.08
Service-oriented	1,199	16.90
Production	3,928	55.38
Other	455	6.41
Years of employment	7,093	17.77 (9.79)

**Table 2:** Sample commuting characteristics and NTL exposure.

Variables	Mean (SD)/proportion
Objective commuting characteristics	
Commute duration (minutes)	23.23 (13.48)
Commute transfers (times)	0.38 (0.95)
Commute cost (yuan)	177.29 (271.55)
Commute flexibility	
Yes	1,393 (19.64)
No	5,700 (80.36)
Subjective commuting characteristics (1–5)	
Crowding	2.58 (1.00)
Discomfort	1.84 (0.95)
Commute-related anxiety	2.19 (1.03)
Unpredictability	2.50 (0.99)
NTL exposure (W/m^2^/sr/μm)	
0 buffer zone	230.12 (478.61)
50 buffer zone	239.15 (360.97)
100 buffer zone	243.05 (358.38)
200 buffer zone	336.74 (422.86)
500 buffer zone	240.68 (216.81)

Regarding commuting characteristics, although substantial heterogeneity was observed across individuals, participants generally reported a moderate level of commuting burden. The average one-way commuting duration was 23.23 min (SD = 13.48), while the mean number of transfers was relatively low (mean = 0.38, SD = 0.95), suggesting that direct commuting routes predominated in the study area. Only a small proportion of participants reported flexible commuting schedules, indicating that most individuals were constrained by fixed commuting times and routes. Subjective commuting experiences further showed relatively high levels of perceived crowding, discomfort, anxiety or irritability, and perceived unpredictability of commuting, which corroborates the multidimensional nature of perceived commuting stress captured by the PCSI.

**Figure 1:**
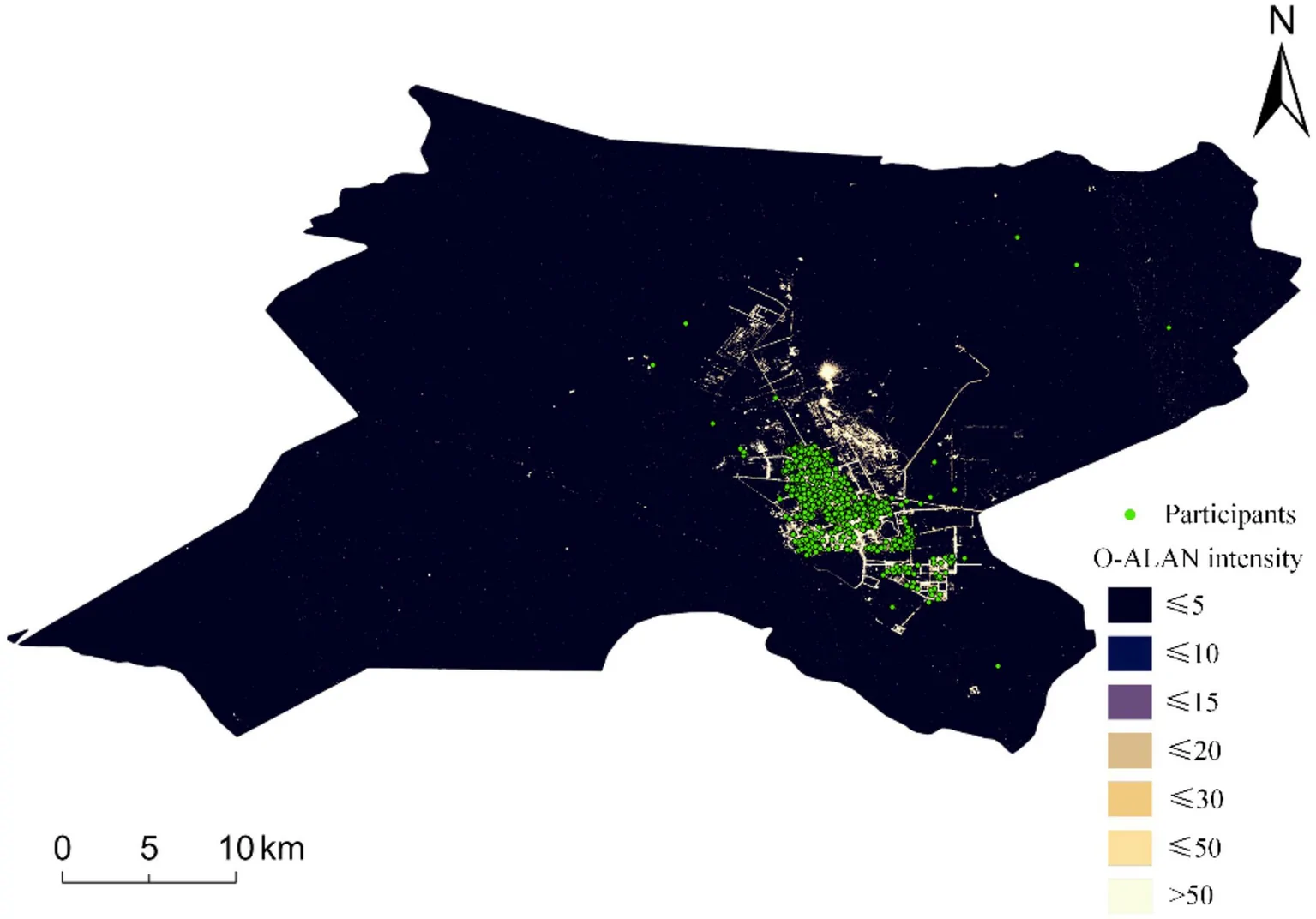
Distribution of residential NTL exposure in Jiayuguan.

Residential NTL exposure also exhibited substantial spatial variability. Differences in NTL intensity were observed across all spatial scales, indicating heterogeneous nighttime environmental conditions within the urban area. Spatial visualization further revealed that higher levels of NTL exposure were primarily concentrated in the urban built-up areas and transportation hubs, whereas peripheral residential zones exhibited relatively lower light intensity (Figure 1).

### 3.2 Correlations between commuting stress and mental health

**Figure 2:**
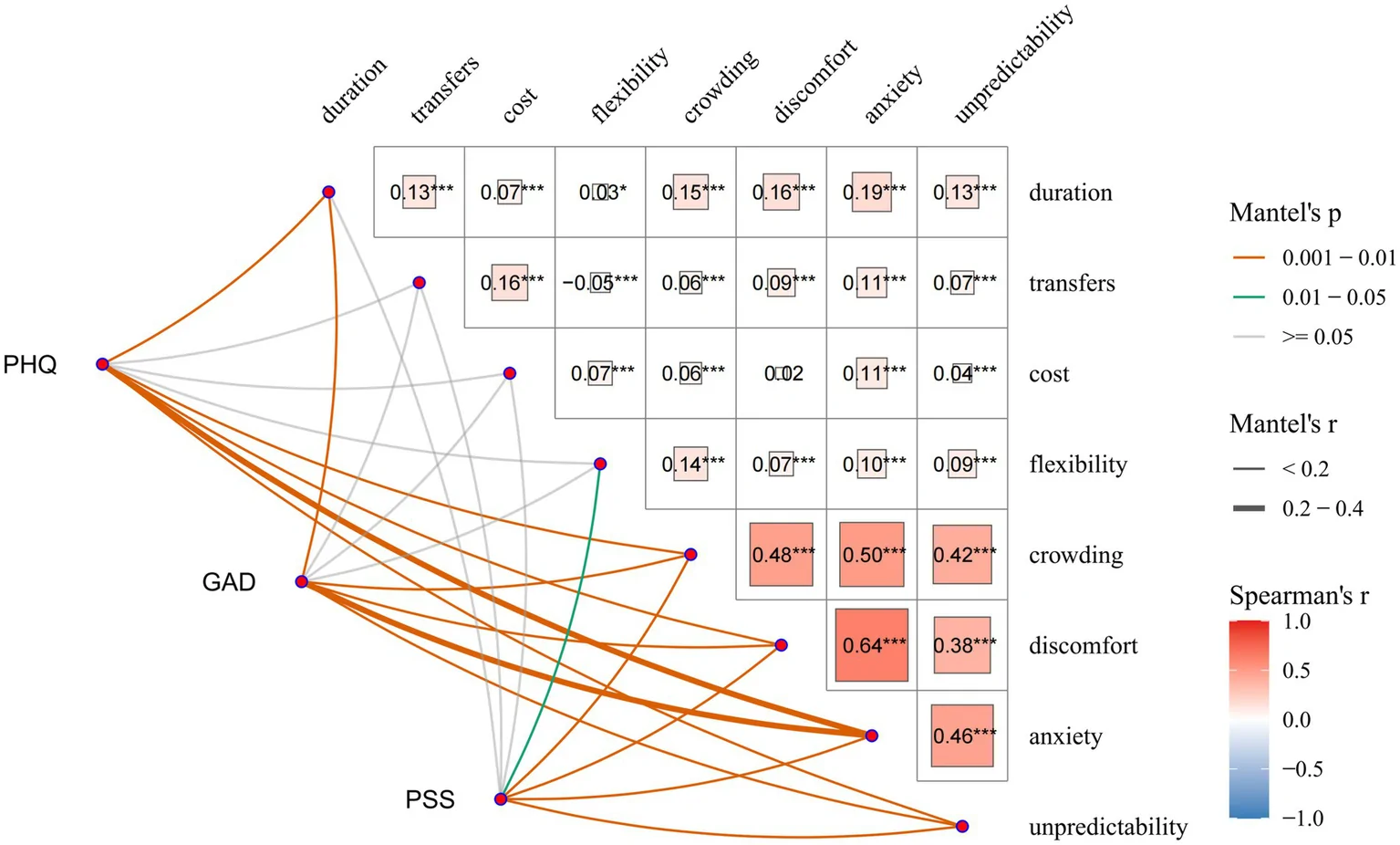
Spearman correlation heatmap of commuting factors integrated with the Mantel test on mental health.

Figure 2 presents the correlation structure between commuting stress-related indicators and mental health outcomes. The network component on the left illustrates the results of the Mantel tests, where the color and thickness of the edges represent statistical significance and the magnitude of Mantel’s r, respectively. The triangular heatmap on the right displays Spearman correlations among commuting environment indicators, with asterisks indicating statistical significance levels.

The Spearman correlation matrix reveals significant associations between objective commuting indicators and subjective stress perceptions. Commuting duration was positively correlated with commuting-related anxiety and physical discomfort. In addition, moderate to strong correlations were observed among subjective stress dimensions, including perceived crowding, discomfort, anxiety, and unpredictability, supporting the internal consistency of the perceived commuting stress construct.

The Mantel test further quantified the overall associations between these stressors and mental health outcomes. Subjective perception variables—particularly commuting anxiety, crowding, and discomfort—emerged as the strongest predictors of psychological distress, showing highly significant and strong associations with both depressive symptoms (PHQ) and anxiety symptoms (GAD). In contrast, objective indicators such as commuting cost and number of transfers exhibited weak or non-significant direct associations with mental health outcomes. Although commuting duration remained statistically significant, its effect size (Mantel’s r) was substantially smaller than that of subjective perception factors, suggesting that perceived commuting stress may serve as a key linking mechanism between objective commuting conditions and mental health outcomes.

### 3.3 Main associations between perceived commuting stress and mental health

We conducted multivariate regression analyses to examine the effects of perceived commuting stress and residential NTL exposure on mental health outcomes, while controlling for sociodemographic, socioeconomic, and occupational covariates. All regression models were estimated using heteroskedasticity-robust standard errors. Table 3 reports the results across different buffer scales for residential NTL exposure.

**Table 3:** Main effects of commuting stress and NTL exposure on mental health outcomes across buffer scales.

Buffer (m)	0	50	100	200	500
PSS					
β_PCSI	3.722444	3.720399	3.721545	3.719114	3.718621
p_PCSI	<0.001	<0.001	<0.001	<0.001	<0.001
β_NTL	0.00205	0.00467	0.00359	0.0039	0.00864
p_NTL	0.2238	0.03683	0.111015	0.041611	0.020973
PHQ					
β_PCSI	2.806031	2.805171	2.805958	2.803581	2.803546
p_PCSI	<0.001	<0.001	<0.001	<0.001	<0.001
β_NTL	0.00113	0.00222	0.00133	0.00265	0.00537
p_NTL	0.382638	0.195634	0.439128	0.07069	0.060503
GAD					
β_PCSI	2.387768	2.387104	2.387258	2.385854	2.386205
p_PCSI	<0.001	<0.001	<0.001	<0.001	<0.001
β_NTL	0.00032	0.00119	0.00113	0.00175	0.00293
p_NTL	0.775933	0.430052	0.45915	0.177203	0.246039

The estimated coefficients for PCSI remained stable across buffer definitions ranging from 0 to 500 meters, and consistently across all three mental health outcomes. In contrast, the estimated effects of NTL showed limited variation across buffer sizes, with statistical significance observed only at larger spatial scales for certain outcomes. This spatial sensitivity highlights the contextual nature of environmental light exposure and underscores the importance of buffer specification when assessing its health impacts.

The multivariate OLS regression results indicate a strong and consistent association between perceived commuting stress and mental health outcomes (Table 4). The PCSI was positively associated with perceived stress, depressive symptoms, and anxiety symptoms, with all associations exhibiting relatively large effect sizes and high statistical significance. After adjusting for a comprehensive set of sociodemographic characteristics, employment attributes, and residential status, these associations remained stable, suggesting that subjective commuting stress is an independent and robust determinant of mental health among employed adults. In contrast, NTL exposure exhibited relatively weaker and outcome-specific main effects. A moderate positive association was observed between NTL and perceived stress, whereas its associations with depressive and anxiety symptoms were attenuated in fully adjusted models and were either marginally significant or statistically non-significant. This pattern suggests that environmental NTL exposure may exert limited direct effects on mental health and is more likely to operate through more nuanced contextual or interactive pathways.

**Table 4:** Main effects of commuting stress and NTL exposure on mental health outcomes.

Variable	PSS		PHQ		*GAD*	
	Coefficient	*p*	Coefficient	*p*	Coefficient	*p*
PCSI	3.7186	<0.001	2.8035	<0.001	2.3862	<0.001
NTL	0.0009	0.021	0.0005	0.061	0.0003	0.246
Sex						
Male	0.3266	0.192	0.745	<0.001	0.5948	<0.001
Age						
25–34	1.6205	0.003	0.8565	0.042	0.9422	0.011
35–44	2.6167	<0.001	1.3053	0.004	1.39	0.001
45–54	2.0435	0.002	0.7985	0.108	1.2297	0.005
≥55	1.2482	0.091	0.2063	0.715	0.5389	0.28
Education						
Middle School	0.6098	0.531	−0.6098	0.413	0.1501	0.82
High School	0.5736	0.535	−0.1097	0.877	0.4109	0.511
Junior College	0.4209	0.648	0.2246	0.75	0.5731	0.357
Bachelor	−0.0647	0.944	0.1455	0.838	0.6574	0.295
Master	−2.4486	0.034	−0.6669	0.449	0.0534	0.945
Marital status						
Married	0.3209	0.407	0.0398	0.893	−0.002	0.994
Divorced	0.2001	0.699	0.5731	0.148	0.1098	0.753
Widowed	2.83	0.031	1.8237	0.069	1.4985	0.09
Number of children						
1	−0.3697	0.277	−0.2725	0.295	−0.0074	0.974
2	−0.2899	0.429	−0.1626	0.562	0.1298	0.6
>3	0.6779	0.484	−0.8461	0.253	−0.3256	0.619
Monthly income						
3,001–5,000	−0.1715	0.688	−0.4544	0.165	0.1199	0.678
5,001–8,000	−0.477	0.274	−0.8615	0.01	−0.1028	0.727
8,001–12,000	−0.8854	0.095	−1.3786	0.001	−0.0989	0.782
12,001–20,000	−0.9492	0.476	−1.4033	0.168	0.0216	0.981
>20,000	1.2719	0.649	1.267	0.553	2.1787	0.248
Hukou-based residential status						
Local_Rural	1.167	0.029	0.2924	0.473	0.3042	0.398
Towns_Outside	−0.2565	0.528	−0.1758	0.572	−0.5463	0.047
Rural_Outside	0.2483	0.314	−0.2908	0.123	−0.167	0.316
Job position type						
Technology	0.8586	0.02	0.6315	0.025	0.3742	0.133
Services	−0.1988	0.607	0.0161	0.956	−0.537	0.04
Production	1.2114	<0.001	1.2008	<0.001	0.4576	0.049
Other	0.1592	0.725	0.1385	0.689	−0.2423	0.428
Employment type						
Outsourced	−0.7016	0.101	−1.4275	<0.001	−0.8491	0.003
Temp	0.3248	0.65	−0.0194	0.972	0.1817	0.708
Freelance	−2.6833	0.175	−0.4694	0.756	−0.3535	0.791
Years of employment	0.0361	0.01	0.0247	0.021	0.0118	0.21

### 3.4 Mediation through perceived commuting stress

**Figure 3:**
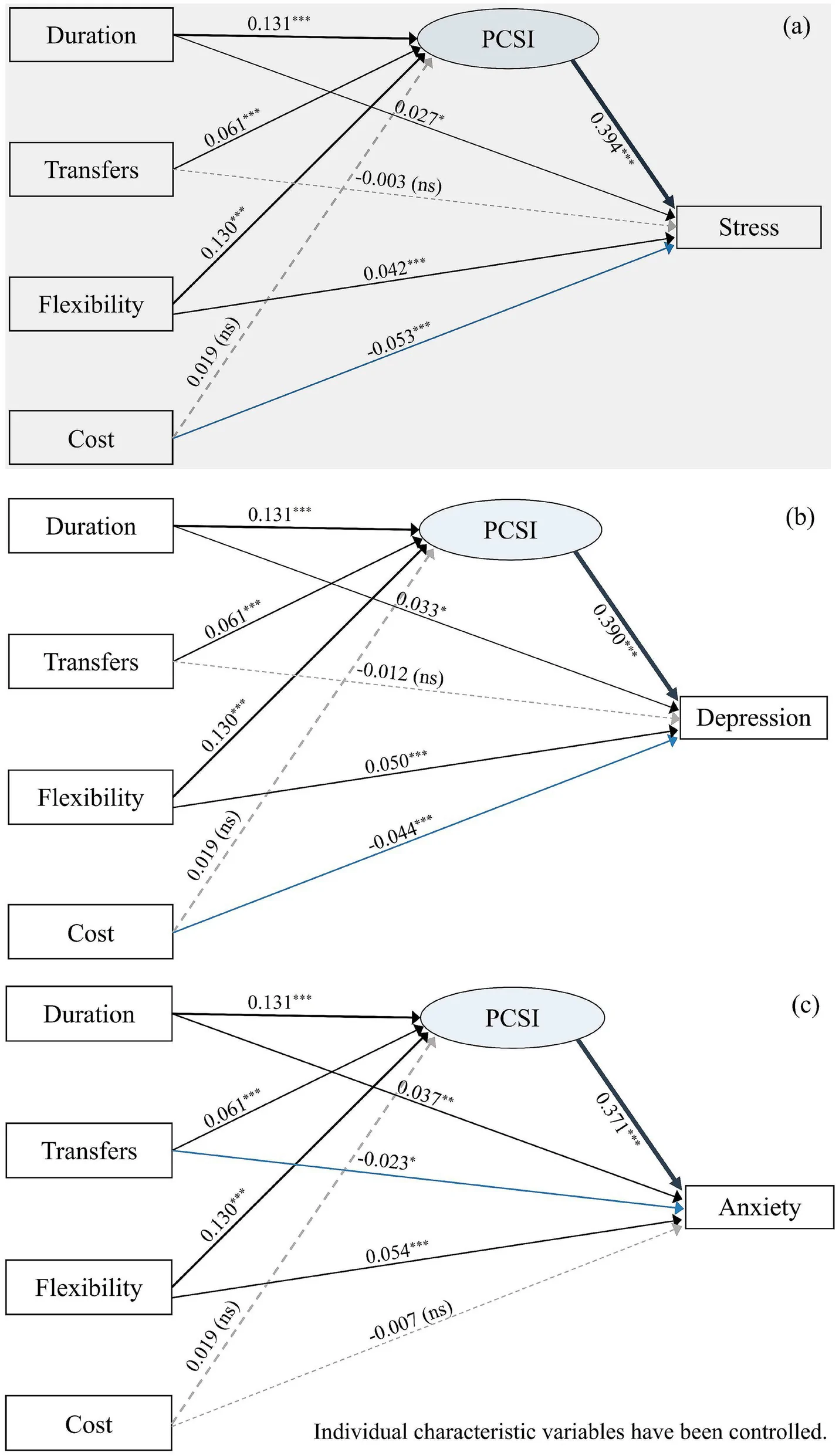
SEM of commuting characteristics and mental health outcomes mediated by perceived commuting stress.

This study employed SEM to examine the mechanisms through which objective commuting characteristics influence employee perceived stress, depression, and anxiety, with the overall mediation framework illustrated in Figure 3. Table 5 reports the total, direct, and indirect effects of objective commuting characteristics on mental health outcomes.

**Table 5:** Decomposition of total, direct, and indirect effects of commuting on mental health.

Variables	Effect	PSS		PHQ		GAD	
		Coef.	95% CI	Coef.	95% CI	Coef.	95% CI
Duration	Total effect	0.591***	[0.372, 0.853]	0.480***	[0.310, 0.685]	0.425***	[0.279, 0.601]
	Indirect Effect	0.387***	[0.282, 0.509]	0.292***	[0.214, 0.383]	0.242***	[0.178, 0.320]
	Direct effect	0.204*	[0.041, 0.399]	0.189*	[0.051, 0.345]	0.183**	[0.061, 0.320]
Transfers	Total effect	0.155	[−0.016, 0.321]	0.068	[−0.064, 0.206]	−0.002	[−0.120, 0.107]
	Indirect Effect	0.180***	[0.110, 0.260]	0.137***	[0.087, 0.195]	0.114***	[0.070, 0.164]
	Direct effect	−0.026	[−0.201, 0.134]	−0.069	[−0.193, 0.057]	−0.116*	[−0.228, −0.015]
Flexibility	Total effect	0.701***	[0.529, 0.874]	0.578***	[0.440, 0.715]	0.513***	[0.404, 0.640]
	Indirect Effect	0.384***	[0.313, 0.458]	0.291***	[0.236, 0.347]	0.242***	[0.197, 0.289]
	Direct effect	0.317***	[0.149, 0.480]	0.287***	[0.151, 0.413]	0.271***	[0.162, 0.385]
Cost	Total effect	−0.342***	[−0.510, −0.149]	−0.213***	[−0.324, −0.091]	−0.003	[−0.113, 0.113]
	Indirect Effect	0.055	[−0.024, 0.140]	0.040	[−0.020, 0.106]	0.033	[−0.016, 0.088]
	Direct effect	−0.397***	[−0.571, −0.227]	−0.253***	[−0.372, −0.142]	−0.036	[−0.137, 0.067]

***p < 0.001, **p < 0.01, *p < 0.05.

Overall, PCSI played a significant and stable mediating role in the relationship between objective commuting characteristics and employee mental health outcomes, revealing heterogeneous mediation patterns across different dimensions of commuting exposure. Across all models, longer commuting duration and lower commuting flexibility were consistently associated with higher levels of perceived commuting stress, which in turn was associated with poorer mental health outcomes. With respect to commuting duration, a substantial proportion of the total association was mediated through PCSI, ranging from 56.9% for anxiety symptoms to 65.5% for perceived stress. A similar pattern was observed for commuting flexibility, for which indirect effects accounted for 47.2 to 54.8% of the total associations across outcomes. These findings suggest that subjective stress appraisal may represent an important psychological pathway statistically linking objective commuting demands with mental health outcomes.

The effects of the number of commuting transfers on different mental health indicators exhibited notable heterogeneity in their pathways. Although the total effects on mental health outcomes were relatively weak, the indirect effects mediated through PCSI were consistently positive and statistically significant across all three outcomes. In the models for perceived stress and depressive symptoms, the direct effects of transfers were not statistically significant, whereas the indirect pathways through PCSI remained significant, suggesting that the association between commuting complexity and mental health appears to be more strongly related to subjective stress appraisal. In the anxiety model, the indirect effect via PCSI remained positive and significant, while the direct effect was negative, resulting in an overall effect close to zero. This pattern indicates the presence of a potential suppression effect.

In contrast, evidence for a mediating role of perceived commuting stress in the association between commuting cost and mental health outcomes was limited. The direct effect of commuting cost on anxiety was entirely non-significant, suggesting that economic burden is not a primary driver of anxiety in this population. Interestingly, commuting cost exhibited a statistically significant negative direct association with both perceived stress and depressive symptoms, indicating that higher commuting expenditures were associated with lower levels of stress and depression. One possible explanation is that higher commuting expenditure may reflect access to more comfortable or efficient transportation modes, although this mechanism was not directly examined.

### 3.5 Moderating effect of NTL

To examine the moderating role of NTL exposure in the association between commuting stress and mental health outcomes, we introduced an interaction term between PCSI and residential NTL exposure in multivariate linear regression models. Prior to constructing the interaction term, both PCSI and log-transformed residential NTL exposure were mean-centered to reduce potential multicollinearity. All models were adjusted for sociodemographic, socioeconomic, and occupational covariates, and heteroskedasticity-robust standard errors were applied. The results are presented in Table 6.

**Table 6:** Moderating role of residential NTL exposure in the association between perceived commuting stress and mental health outcomes.

Variables	PSS	PHQ	GAD
	Coef. (95% CI)	Coef. (95% CI)	Coef. (95% CI)
PCSI	3.62*** (3.41, 3.83)	2.71*** (2.55, 2.87)	2.33*** (2.18, 2.48)
NTL	0.0007* (0.0001, 0.0013)	0.0004 (−0.0000, 0.0009)	0.0002 (−0.0003, 0.0007)
PCSI × NTL	0.29** (0.12, 0.46)	0.18* (0.02, 0.41)	0.07 (−0.06, 0.20)
Covariates	Yes	Yes	Yes
Observations	7,093	7,093	7,093
*R* ^2^	0.236	0.238	0.220

Regression models were estimated using heteroskedasticity-robust standard errors. Residential NTL was log-transformed and subsequently standardized using z-scores prior to analysis. The PCSI was also standardized before constructing interaction terms. Interaction models included all covariates shown in the table. ***p < 0.001, **p < 0.01, *p < 0.05.

The interaction analyses indicate that NTL exposure plays a significant positive moderating role in this relationship, although its effects vary across different mental health outcomes (Table 6). Specifically, the interaction term between PCSI and residential NTL exposure was positively associated with perceived stress, suggesting that the association between perceived commuting stress and perceived stress is stronger under conditions of higher residential NTL exposure. A similar moderating pattern was observed for depressive symptoms, albeit with a relatively weaker magnitude. Although the direct association between residential NTL exposure and depressive symptoms was small and statistically non-significant, higher NTL exposure was associated with a stronger positive association between perceived commuting stress and depressive symptoms.

For anxiety symptoms, the interaction term was positive but not statistically significant, indicating that the moderating effect of NTL on the relationship between PCSI and anxiety is weak or limited. Overall, these findings suggest that residential NTL exposure functions primarily as a contextual condition rather than an independent environmental stressor.

## 4 Discussion

This study examined how objective commuting demands, perceived commuting stress, and residential NTL exposure are jointly associated with mental health of employed adults in an industrial city. Overall, the findings are consistent with the environmental stress appraisal framework: objective commuting conditions are primarily associated with mental health through subjective commuting stress, whereas residential NTL exposure appears to operate mainly as a contextual environmental condition associated with stronger stress-related psychological responses. Rather than acting as a stable and independent risk factor, residential NTL exposure shapes the broader nighttime environmental context in which daily stress and recovery processes occur. By distinguishing between objective commuting demands and subjective stress appraisal, this study extends the existing commuting literature beyond an exposure-centered paradigm toward a more integrative framework, highlighting the critical role of psychological perception mechanisms in understanding how everyday environmental demands affect mental health. In addition, by linking commuting stress appraisal with spatially measured nighttime residential environments, the study contributes to environmental psychology by demonstrating that daily stress experiences may also be related to the environmental context surrounding post-work recovery processes.

The results suggest that perceived commuting stress may serve as a more proximal psychological mechanism linking commuting experiences to mental health than objective commuting characteristics alone. Although prior studies have typically used commuting time, commuting distance, or mode of transport as indicators of commuting burden and have examined their direct associations with stress levels, depressive symptoms, or subjective well-being (Ettema et al., 2010; Stutzer and Frey, 2008), such exposure–outcome relationships are often unstable and may be moderated by individual psychological processes. The environmental stress appraisal framework provides a coherent explanation for this pattern: environmental demands do not automatically translate into psychological strain; rather, their effects depend on how individuals cognitively and affectively evaluate these experiences (Lazarus and Folkman, 1984).

In the commuting context, although objective commuting characteristics are associated with stress responses and subjective well-being, emotional reactions and appraisal processes play a central mediating role in these relationships (Murphy et al., 2023). Evidence from panel data further indicates that the adverse effects of longer commuting duration on mental health are largely transmitted indirectly through subjective commuting experiences and their spillover effects on leisure and family life (Clark et al., 2020). Recent empirical studies using time-stamped subjective and objective measures also reveal discrepancies between physiological stress responses and self-reported stress during commuting, suggesting that subjective appraisal may more directly capture the pathway through which psychological stress operates (Zhang and Ma, 2024). Taken together, research on commuting and mental health may benefit from shifting its focus away from commuting exposure per se toward the process through which commuting experiences are appraised as psychological stress. From an environmental psychology perspective, commuting is not merely a transportation activity, but a recurrent environmental demand embedded within broader urban daily life. Accordingly, interventions aimed at alleviating commuting-related psychological burden should not be limited to reducing commuting time or distance; rather, they should pay greater attention to how commuting environments are perceived and experienced in everyday life, including factors such as predictability, comfort, perceived control, and emotional strain.

The direct association between residential NTL exposure and depressive and anxiety symptoms is relatively limited; however, it exhibits a significant moderating effect on the relationship between perceived commuting stress and mental health. Rather than functioning as an independent environmental risk factor, NTL exposure may be better conceptualized as a form of contextual environmental load, which shapes the external conditions under which daily stress coping and psychological recovery occur.

On one hand, previous studies suggest that certain lighting characteristics may be associated with circadian regulation and recovery-related processes, thereby attenuating the typical emotional rebound and stress buffering that would occur after commuting, and potentially exerting a protective effect against depression and anxiety (Hu et al., 2025). The underlying physiological explanation is that warm light, compared with cool light, may facilitate increased nighttime melatonin secretion, thereby influencing circadian regulation (Xiao et al., 2021). On the other hand, excessive and continuous NTL can disrupt circadian rhythms, interfering with sleep–wake behavior, hormone secretion, cellular function, and gene expression. In particular, abnormal light exposure can alter the functioning of brain regions involved in emotion and affect regulation, triggering depressive symptoms (Bedrosian and Nelson, 2017). Under high commuting stress, individuals’ emotional regulation capacity and psychological resilience are partially depleted, increasing their sensitivity to environmental stimuli. Notably, the NTL data in this study, derived from remote sensing, do not differentiate specific types of lighting or color temperature, but rather serve as an integrated measure of NTL pollution intensity. In such contexts, NTL is no longer a neutral environmental background; for individuals already experiencing elevated stress, it acts as an adverse factor by enhancing fatigue, irritability, and cognitive load, which may be consistent with reduced opportunities for psychological recovery (Helbich et al., 2024; Shao et al., 2025).

The observed moderating pattern for NTL is consistent with the pathway of “objective environmental exposure—subjective discomfort experience—mental health outcomes.” As a measurable environmental feature, nighttime lighting may be related to individuals’ emotional experiences and perceived stress by altering their perceived surroundings, which can cumulatively contribute to adverse mental health outcomes. The relationship between NTL intensity and residential comfort is complex and nonlinear. A nationwide survey in Japan demonstrated that when the average community-level net light pollution exceeds 10 nW/cm^2^/sr, residential comfort significantly declines (Li and Managi, 2023). Research conducted in Beijing further indicated that in areas with high illumination, individuals are more sensitive to the uniformity, safety, and comfort of lighting (Wei et al., 2022). Such discomfort can heighten depressive tendencies and reduce resilience against environmental stressors. Importantly, not all individuals’ needs are fully captured by average lighting conditions; personal preferences and expectations can influence how lighting environments are perceived and evaluated, with positive expectations sometimes exerting a greater effect on subjective experience than illuminance itself (Maier et al., 2017). Collectively, these findings indicate that environmental exposure impacts mental health through multifaceted pathways, heavily mediated by subjective perception and psychological regulation, with NTL serving as a contextual environmental condition within this process.

As a component of the urban environment, our findings on nighttime lighting can be extended to a broader framework linking the urban environment to mental health via the core logic of “objective exposure—subjective experience—health outcomes.” Rather than treating urban environmental factors as independent risk sources, our results emphasize their amplifying and moderating roles within stress contexts. Various elements of the urban environment operate as an interconnected system, mutually influencing one another and affecting the psychological and neurobiological characteristics of urban residents, ultimately alleviating or exacerbating the burden of common mental disorders (van der Wal et al., 2021). Neurobiological research further indicates that environmental risk factors can disrupt neural circuits associated with negative emotions, stress, and salience regulation, thereby aggravating psychiatric symptoms (Meyer-Lindenberg and Tost, 2012). In this sense, our study offers a context-dependent, process-oriented extension to urban environment–mental health theory, suggesting that the health effects of environmental exposure are not fixed but are contingent upon individuals’ stress levels and subjective perception processes.

Furthermore, this study highlights the significant advantages of using remote sensing data to characterize urban environmental features and investigate their links to mental health. Remote sensing can provide more comprehensive and temporally responsive information on environmental exposures, thereby enriching both the breadth and depth of research on the mechanisms connecting urban environments to mental health (Liu et al., 2020; Tian et al., 2023). However, remote sensing is not intended to replace individual-level surveys or psychometric instruments; rather, it serves as an important complement that can be integrated into process-based models of psychological stress and emotional responses. For example, in this study, NTL exposure is conceptualized as a contextual variable whose health effects depend on individuals’ stress states and subjective perception processes. This demonstrates that remote sensing indicators can not only reveal spatial correlations among variables but also support mechanism-oriented empirical research, providing process-level evidence to explain how environmental factors influence mental health.

The findings of this study have several implications for understanding urban environments and commuting experiences in the context of mental health promotion and healthy cities research. The results suggest that commuting-related psychological burden is not determined solely by objective indicators such as duration or distance, but is more strongly shaped by subjective commuting experiences. Factors such as predictability, perceived control, comfort, and environmental strain may therefore represent key leverage points for improving the daily experiences of employed adults. In addition, residential nighttime environments may serve as an important contextual background for post-work recovery. High-luminance urban environments may be less conducive to psychological detachment and recovery-related experiences after work, highlighting the relevance of nighttime urban conditions not only for urban vitality and safety but also for restorative well-being. More broadly, these findings emphasize the importance of incorporating subjective environmental experiences into urban mental health research, as the health effects of environmental conditions are likely to operate through ongoing stress processes and individual appraisal.

This study has several limitations. First, its cross-sectional design precludes causal inference; future research should adopt longitudinal or quasi-experimental approaches to better establish temporal relationships among commuting stress, nighttime exposure, and mental health. Second, the sample is drawn from trade union members, which may limit generalizability due to potential socioeconomic and occupational selection bias. Third, the NTL data capture only aggregate intensity and cannot distinguish between specific light sources; future studies could use higher-resolution remote sensing or ground-based data to better differentiate environmental components. Finally, integrating additional environmental exposures and physiological indicators, as well as examining individual differences such as stress susceptibility, would help clarify the mechanisms underlying urban stress processes.

## 5 Conclusion

This study, grounded in the environmental stress appraisal perspective, examined how commuting stress and the urban nighttime environment are associated with the mental health of employed adults. The findings suggest that rather than conceptualizing commuting solely as a direct consequence of objective conditions, its mental health burden is more appropriately understood as a stress appraisal process. Objective commuting demands were associated with mental health primarily through perceived commuting stress, highlighting the central role of subjective appraisal in linking everyday environmental demands to psychological outcomes. In addition, the direct association between residential NTL exposure and mental health was relatively limited; however, it appeared to operate as a contextual moderator under high-stress conditions, with stronger associations between commuting stress and adverse mental health outcomes. By integrating satellite-derived environmental indicators with individual psychological assessments, this study demonstrates that combining spatially measured environmental conditions with subjective evaluations provides a viable methodological approach for investigating everyday urban stress within environmental psychology.

## Data Availability

The individual-level survey data used in this study are not publicly available because they contain sensitive information on mental health, commuting experiences, sociodemographic characteristics, and geocoded residential exposure. Data sharing is restricted by ethical approval conditions and data-use agreements with the collaborating organization. Participants did not provide consent for public deposition or unrestricted secondary use of the raw or derived individual-level datasets. Therefore, the datasets cannot be uploaded to a public repository or made available by request to the corresponding author. Aggregated results supporting the findings are reported in the article.
